# A computational study of the quantum transport properties of a Cu–CNT composite†
†Electronic supplementary information (ESI) available. See DOI: 10.1039/c5cp01470k



**DOI:** 10.1039/c5cp01470k

**Published:** 2015-06-29

**Authors:** Mahdi Ghorbani-Asl, Paul D. Bristowe, Krzysztof Koziol

**Affiliations:** a Department of Materials Science and Metallurgy , University of Cambridge , Cambridge , CB3 0FS , UK . Email: mg741@cam.ac.uk ; Email: pdb1000@cam.ac.uk

## Abstract

The quantum transport properties of a Cu–CNT composite are studied using a non-equilibrium Green's function approach combined with the self-consistent-charge density-functional tight-binding method. The results show that the electrical conductance of the composite depends strongly on CNT density and alignment but more weakly on chirality. Alignment with the applied bias is preferred and the conductance of the composite increases as its mass density increases.

## Abstract 2

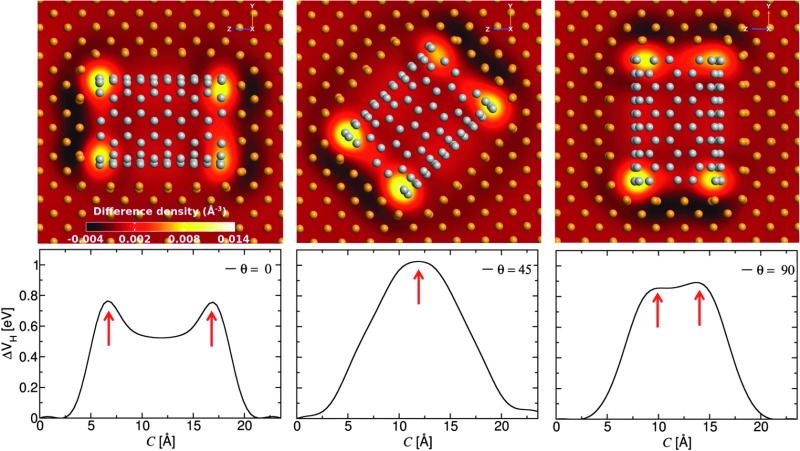
Electron difference density maps through cross-sections of three differently oriented Cu–CNT (6,6) composites (top) and the electrostatic difference potential along the transport direction (bottom).

The growing demand for electrical energy, changing weather conditions, rising prices and the need for power in extreme environments all limit the use of conventional conductors in electrical wires and interconnects. Furthermore, power losses cause serious performance issues in these conductors. Novel composite materials containing carbon nanostructures can potentially improve a number of properties of conventional conductors but one important area would be to allow them to transmit current more efficiently and at lower cost. They would also reduce the weight required for a designated transmission line. Carbon nanotubes (CNTs) are obvious candidates for this purpose because of their remarkable physical properties, *e.g.* high current carrying capacity,^1^ ballistic transport,^2^ low density, and high strength and stiffness.^3–5^ There have already been reports of the promising electrical properties of high quality CNT-based fibres.^6,7^ The fibres have also been used in various electrical applications, *e.g.* high frequency transformers.^8^ Although CNTs on their own exhibit low free electron density,^9^ this can be addressed by embedding them in transition or simple metals like Cu, Cr, Au, Ag or Al to form novel composite materials with high conductivity.^10^ Among these possibilities, Cu–CNT composites have attracted particular attention because of their excellent electrical conductivity^11^ (2.3 – 4.7 × 10^5^ S cm^–1^ compared to 5.8 × 10^5^ S cm^–1^ for bulk copper), high thermal conductivity^12^ (395 Wm^–1^ K^–1^ compared to 400 Wm^–1^ K^–1^ for bulk copper) and low coefficient of thermal expansion (similar to silicon).^12^ In addition, it is found that Cu–CNT composites are about three times stronger than pure Cu.^13^ Such composites can be fabricated using various techniques involving powder metallurgy, thermal spraying and electrochemical deposition^10^ although the results show that the electrical conductivity depends strongly on the fabrication process. It is expected that better control over the structural morphology of the CNTs will result in improvements to the conductivity. Computational analysis of the underlying transport properties and microstructure of the composites will guide this process.

First principles calculations on the interaction between copper and CNTs have so far focused on the end or side contact between a nanotube and two copper electrodes.^14–17^ Electron transport across the interface between the CNT and the metal surface is clearly important but the CNT is not embedded within the metal and therefore other transmission pathways cannot occur. More recently, Feng *et al.* ^18^ studied the electrical performance of a Cu–CNT matrix using a simple electrical model and concluded that the ratio of aligned CNTs and their dimensions affect the current density distribution. To gain further insight at the microscopic level large-scale quantum transport calculations are necessary. In this work, the electronic structure and transport properties of Cu–CNT composites are investigated using a density-functional based approximation method combined with the non-equilibrium Green's function (NEGF) scheme. The effect of CNT density, chirality and alignment to the bias potential is studied. Due to the presence of quantum confinement, each parameter is likely to influence the electrical properties. For the first time a model is constructed in which the CNT is fully embedded within a 3D copper matrix. The results show that the conductance is highest when the long axis of the CNT is parallel to the applied bias. Furthermore the conductance decreases with CNT number density and increases with overall mass density of the composite as expected.

The atomistic model used in the calculations is shown in Fig. 1 and includes a finite central or scattering region, which is connected to two semi-infinite ideal copper electrodes across which a bias is applied. In the scattering region the CNT, which is empty, is contained within the Cu matrix and therefore both the curved surface and ends of the CNT participate in the electron transport. Periodic boundary conditions are applied in all directions perpendicular to the applied bias. Structurally there are many ways to orient the CNT within the metal matrix. In order to maintain symmetry and reduce complexity, three principal configurations are studied with *θ* = 0°, 45°, 90° where *θ* is the angle of inclination of the nanotube axis with respect to the transport direction (the horizontal *c*-axis in Fig. 1). Most of the calculations are performed using a rectangular supercell with dimensions 14.8 Å, 23.6 Å and 23.6 Å along *a*, *b* and *c* directions. This corresponds to a CNT number density of 2.87 × 10^13^ cm^–2^. To investigate composites with different CNT densities the dimensions along *a* and *b* are changed. The largest supercell has dimensions 14.8 × 39.0 × 23.6 Å corresponding to 1080 atoms and a CNT number density of 1.73 × 10^13^ cm^–2^. Both a metallic CNT (6,6) and a semiconducting CNT (10,0) with almost same length (∼11 Å) and very similar diameter (∼8 Å) are considered. This enables a direct comparison of the results for these two cases without the complication of the size dependence. The volume of the Cu cavity containing the CNT is chosen such that the distance between the Cu and CNT surfaces agrees with recent density functional theory (DFT) results^15,16^ for optimal separation between the materials, *i.e.* about 1.9–2.4 Å. For the *θ* = 0° and 90° orientations, the CNT connects with Cu{100} surfaces at the end contacts whereas for the *θ* = 45° orientation the surfaces are {110}. To further support this choice of orientations and surface contacts, separate DFT calculations were performed on a (5,5) CNT in contact with three low-index Cu surfaces (see Table S1, ESI†). A (5,5) CNT is chosen for computational convenience and no significant difference in behaviour is expected if a (6,6) CNT is used. It is found that the {100} orientation is the most stable for both the side and end contacts suggesting they have the lowest energy. The {110} end and side contacts are found to be less stable but by no more than 6% of the ideal work of separation.

**Fig. 1:**
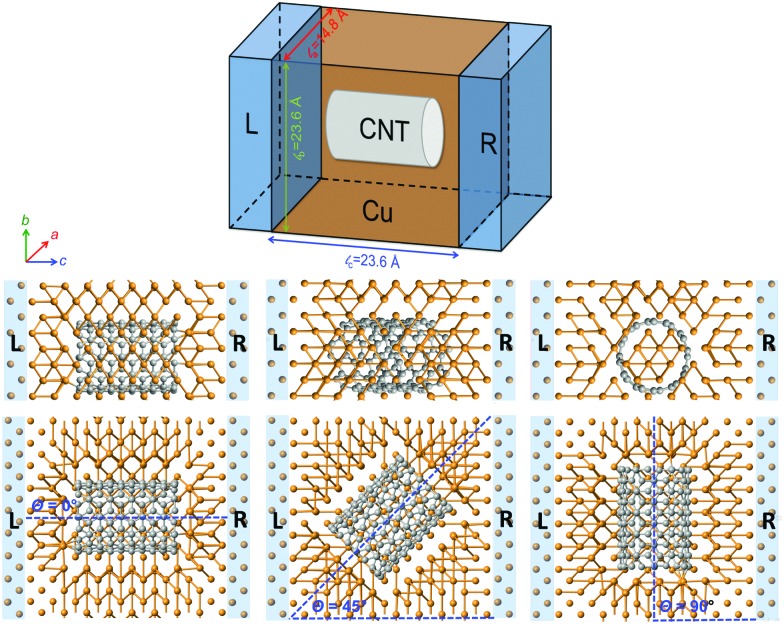
Upper: Schematic representation of the model used for the Cu–CNT transport calculations. Lower: Optimized structure of a Cu–CNT composite containing a (6,6) CNT in three different orientations (*θ*) with respect to the transport direction (horizontal *c*-axis). In each case a top and side view is shown. The left and right electrodes (L and R) consist of semi-infinite perfect copper. Carbon and copper atoms are shown in grey and orange, respectively.

All the composite calculations are performed using the density-functional-based tight-binding (DFTB) method^19^ as implemented in the Atomistix ToolKit (ATK).^20^ The structures are optimized applying 3D periodic boundary conditions and total energies are converged to within 10^–5^ eV. The Brillouin zone of the whole structure is sampled using (1 × 1 × 100) *k* points. The Poisson equation is solved using a Fast Fourier Transform (FFT) solver. The coherent transport calculations are performed using the DFTB method in conjunction with the NEGF technique.^21^ The calculations involve the self-consistent charge correction (SCC),^22^ which takes into account the electron density redistribution due to interatomic interactions. The NEGF + DFTB method is an accurate and efficient method for calculating transport properties and has been applied successfully to various nanostructured systems.^23–25^ The DFTB parameters for carbon and copper have been validated and reported earlier.^26^ The steady-state electrical current through the device under non-equilibrium conditions, *i.e.* for a finite bias voltage (*V*_Bias_) is calculated using the Landauer formula:^27^ 
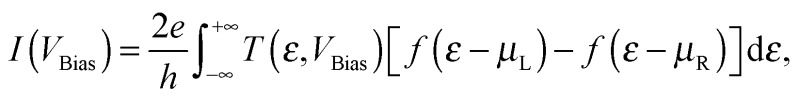
where *f* is the Fermi–Dirac distribution function, 
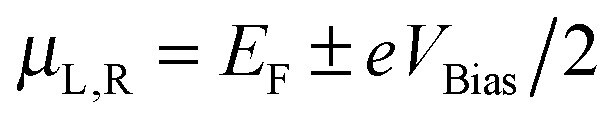
 represent the chemical potentials of the left and right electrodes and *T*(*ε*,*V*_Bias_) is the energy and voltage-resolved transmission function. Electron transport is assumed to be ballistic since the electron mean free path in copper (∼40 nm) is much greater than the supercell dimensions. The calculations of the current are performed at 300 K. For reference the bulk atomic and electronic structures of copper and graphene calculated with the DFTB method have been compared with DFT calculations and experimental data and the agreement is good (see Table S2 and Fig. S1, ESI†).

Fig. 2 compares the current–voltage characteristics for composites with different chirality and orientation for models containing 2.87 × 10^13^ cm^–2^ CNTs. Also shown is the characteristic of pure Cu. Note that inelastic scattering effects, *e.g.* electron–phonon coupling, have been ignored in the transport model. However, this approximation does not change the general trends and conclusions obtained here, as the relative change in the conductance is not affected. It is seen that the presence of a CNT within the Cu matrix reduces the conductance (slope of the IV curve) by ∼30% in all cases compared to the intrinsic conductance of the copper. Noticeably the current through the composites is suppressed with increasing *θ*. These results are consistent with previous reports that show that the end rather than the side of the CNT is the more favourable electron injection path.^14,15^ The effect of chirality on the conductance, at least for this CNT density, is very small and therefore the rest of the paper will focus on results for composites containing metallic (6,6) CNTs.

**Fig. 2:**
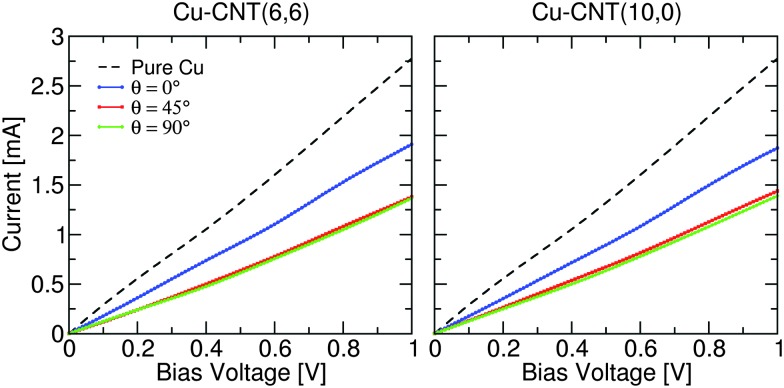
Current–voltage characteristics of Cu–CNT composites with different chirality and orientation. The characteristic of infinite pure Cu is also shown for comparison.

In order to understand how the surface interactions between Cu and the CNT affect the transport properties, the electron difference density and electrostatic difference potential for the three (6,6) CNT orientations are computed (see Fig. 3). The electrostatic difference potential is obtained by solving the Poisson equation where the charge density is taken from the electron difference density. The dark and light areas on the map correspond to electron loss and electron gain, respectively. The charge accumulation is mainly concentrated at the ends of each CNT while there is only slight charge transfer along the side contacts. This agrees well with previous studies on Cu/CNT interfaces.^15^ It can be seen that the presence of the interface causes energy level re-alignment and the formation of a potential barrier, which can quench the electron transport. This evident from the two sharp peaks in the electrostatic difference potential which appear at *c* = ∼6 Å and ∼17 Å for the composite where the CNT is aligned along the bias direction. For the composite with oblique CNT alignment (*θ* = 45°), the two end effects overlap because of the shortness of the nanotube and only one peak is observed which decays smoothly along the transport direction.

**Fig. 3:**
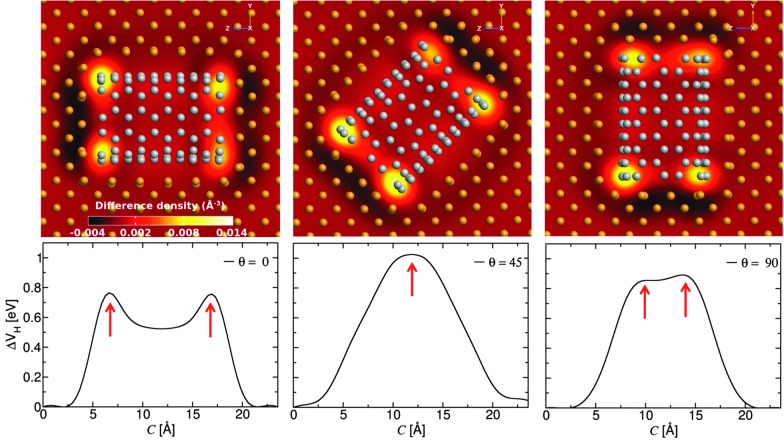
Electron difference density maps through cross-sections of the scattering region (upper) and the average electrostatic difference potential along the transport direction (lower) for the three Cu–CNT (6,6) composite orientations considered. The peak positions are indicated with red arrows to highlight the good correspondence between the upper and lower panel.

Fig. 4(a) shows the projected density of states (PDOS) for the Cu–CNT (6,6) composite with *θ* = 0°. Due to the strong metallic character of the Cu, the electronic structure close to the Fermi level is mostly governed by Cu d orbitals, while C atoms have no significant impact on the total DOS. Because of the dominance by Cu, the orientation of the CNT does not have a significant effect on the total density of states. Further DFT results, shown in Fig. S2 (ESI†), on a Cu/CNT interface indicate that for the end contact the cohesion between the CNT and copper surface is mainly due the coupling between carbon p orbitals and copper d orbitals. For the side contact, some slight sp^2^/sp^3^ re-hybridization of carbon atoms at the interface is seen. The calculated transmission coefficients of the composites for different values of *θ* and bias voltage are shown in Fig. 4(b). The results indicate a strong dependence of the transmission coefficient on the CNT orientation. The transmission value at the Fermi level is highest for *θ* = 0°, while it decreases by about 40% for *θ* = 45° and *θ* = 90°. The fewer available transmission pathways for electrons to flow from the Cu surface to the CNT can rationalize this effect. The overall transmission features are similar for the three cases and they indicate completely delocalized transport channels within the energy range shown here. The transmission coefficients at the Fermi energy are hardly affected by the bias voltage. However, for electron energies lower than the Fermi energy an increase in bias to *V*_Bias_ = 1.0 V leads to a significant decrease in the transmission coefficients. This suggests that electron transport through the composite is much stronger at lower bias than at higher bias. A similar behaviour is observed for composites with a (10,0) CNT (see Fig. S3 and S4, ESI†). The SCC-DFTB transport results also showed very good agreement with DFT results obtained for Cu–CNT composites with the same chirality and orientation (see Fig. S5, ESI†).

**Fig. 4:**
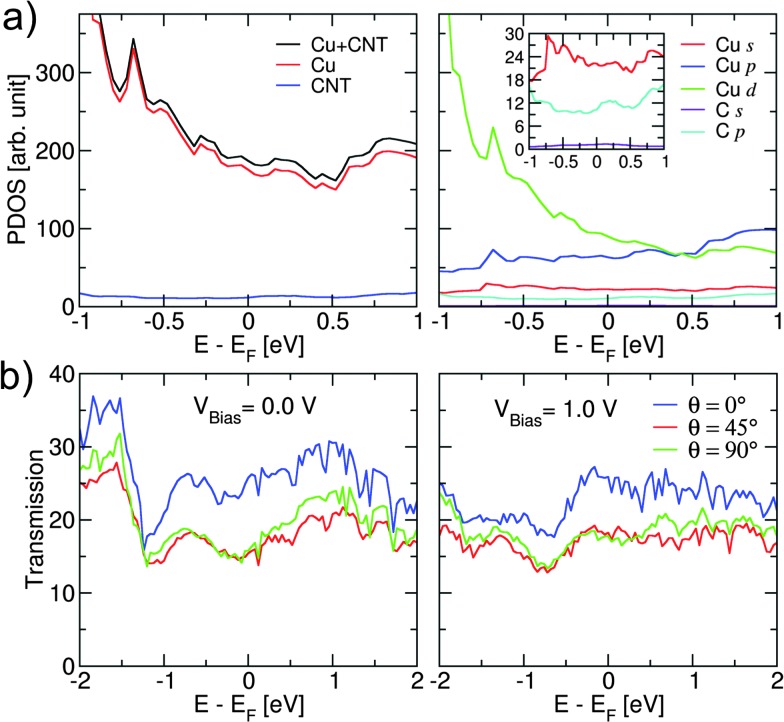
(a) Projected density of states (PDOS) of the Cu–CNT (6,6) composite with *θ* = 0°(see Fig. 1). The inset shows an enlarged view near the Fermi level. (b) Transmission coefficients as a function of energy at *V*_Bias_ = 0.0 V and *V*_Bias_ = 1.0 V for Cu–CNT (6,6) composites with different values of *θ* (see Fig. 1). The Fermi level *E*_F_ is shifted to zero.

To further investigate the transport behaviour in the composite, a comparison is made between the transmission eigenstates and pathways for pure Cu and the Cu–CNT (6,6) composite with the most favourable orientation, *θ* = 0° (see Fig. 5). The results show that the presence of the CNT significantly changes the transmission modes in the composite particularly close to the Cu/CNT interface. The amplitudes of the transmission coefficients are highest around the CNT circumference due to the excellent transport properties of CNT. In addition, the calculated transmission pathways show that some scattering occurs at the Cu/CNT interface that can be responsible for the reduction in transmission through the composite.

**Fig. 5:**
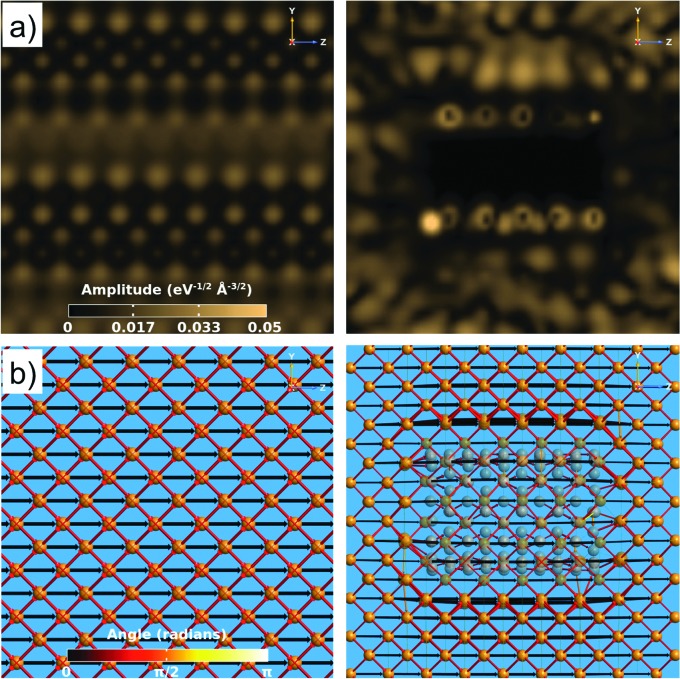
(a) Transmission eigenstates and (b) transmission pathways for pure Cu (left) and a Cu–CNT (6,6) composite with *θ* = 0° (right).

Finally, the specific differential conductance (d*I*/d*V*) of the Cu–CNT (6,6) composite with *θ* = 0° is calculated as a function of CNT number density per unit area and mass density of the composite (see Fig. 6). The highest CNT density per unit area considered in this work is about 5.1 × 10^13^ cm^–2^. This value is consistent with recent theoretical^18^ and experimental data^28^ that report CNT densities around 10^13^–10^14^ cm^–2^ for CNT-based composites. The calculated density of copper (8.4 g cm^–3^) is smaller than the expected value of 8.9 g cm^–3^ due to the ∼2.4% larger DFTB-predicted lattice constant compared to experiment (see Table S2, ESI†). It is seen that conductance decreases with CNT number density and increases with overall mass density of the composite as expected. So, for example, a ∼10% reduction in mass density results in a ∼30% reduction in conductance.

**Fig. 6:**
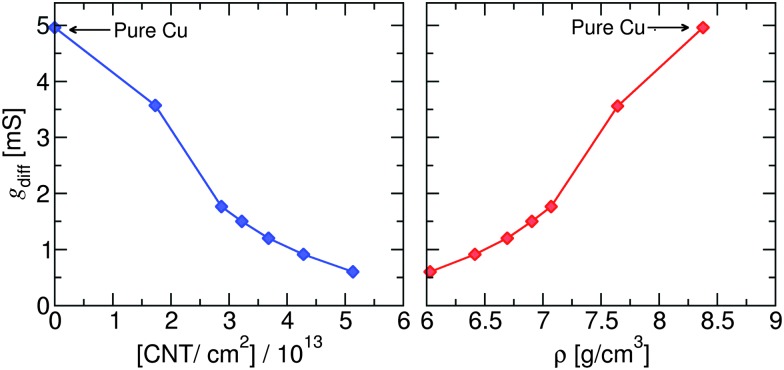
Differential conductance of the Cu–CNT (6,6) composite with *θ* = 0° as a function of CNT number density per unit area (left) and overall mass density of the composite (right).

## Conclusions

Using the non-equilibrium Green's function approach, the transport properties of a Cu–CNT composite are determined as a function of CNT density, chirality and alignment to the bias potential. A model is used in which the CNT is fully embedded within the Cu matrix. The electrical conductance is found to be highest when the axial direction of the CNT is along the transport direction. Compared to pure Cu the reduced conductance is attributed to localized charge exchange between the CNT and the metal surface that raises the potential barrier at the interface. The chirality of the CNT does not appear have a major effect on the conductance at least for the CNTs and densities considered here. The results could play an important role in guiding the design and fabrication of Cu–CNT composites for optimal performance. Understanding and controlling the interface between the Cu and CNT in the bulk composite is critical for maximising the electrical performance of this promising new generation of materials.
