# Supplementary material for: A computational study of the quantum transport properties of a Cu–CNT composite
Source: Phys Chem Chem Phys. 2015 Jun 29;17(28):18273–7. doi: 10.1039/c5cp01470k (PMC4598852; doi:10.1039/c5cp01470k)
Supplement: Supplementary file 1 [file CP-017-C5CP01470K-s001.pdf]

Electronic Supplementary Material (ESI) for Physical Chemistry Chemical Physics.
This journal is © the Owner Societies 2015

A computational study of the quantum transport properties of a Cu-CNT composite

Mahdi Ghorbani-Asl, Paul D. Bristowe and Krzysztof Koziol

Department of Materials Science and Metallurgy, University of Cambridge, Cambridge, CB3 0FS, UK.

Supplementary Information

Our density functional theory (DFT) calculations were performed using the PBE (Perdew-Burke-Ernzerhof) exchange-correlation functional[1] and double-zeta plus polarization basis set of SIESTA-type numerical orbitals as implemented in the ATK package[2]. London dispersion interactions were included in the total bonding energy as proposed by Grimme[3].

The interfacial strength[4] (ideal work of separation) has been calculated using:

$$W_{sep} = \frac{E_{Metal} + E_{CNT} - E_{Metal+CNT}}{L}$$

where E_{Metal} , E_{CNT} and $E_{\mathrm{Metal+CNT}}$ represent the total energies of the isolated metal, the isolated CNT and the metal-CNT contact, respectively. L stands for the contact length.

Table S1. Interfacial strengths (W_{sep}) of different Cu/CNT (5,5) interfaces from DFT calculations.

Type of CNT contact	End Contact			Side contact		
Cu Surface	(100)	(110)	(111)	(100)	(110)	(111)
W_{sep} (eV/Å)	1.227	1.153	1.196	0.983	0.980	0.821

For contact with either the end or side of the CNT, the (100) Cu surface gives the most stable (strongest) interface.

Table S2. Experimental and calculated lattice parameters of copper and graphene obtained at the DFTB and DFT level. Units are given in Å.

Material	Experiment	DFT	DFTB
Copper	*a*=*b*=*c*=3.61	*a*=*b*=*c*=3.67	*a*=*b*=*c*= 3.70
Graphene	*a*=*b*=2.46	*a*=*b*=2.48	*a*=*b*=2.47

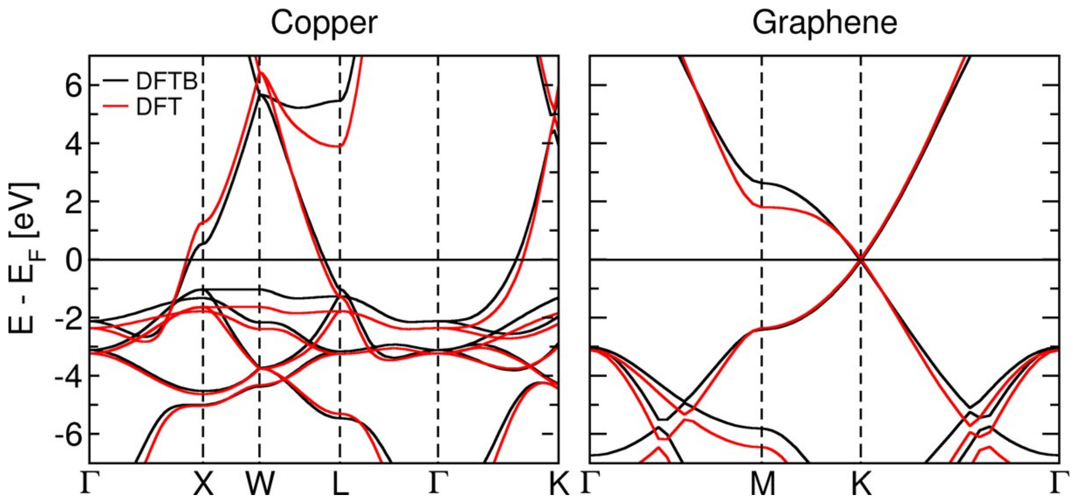

Figure S1. (Colour online) DFT and DFTB electronic band structures calculated for bulk copper and graphene.

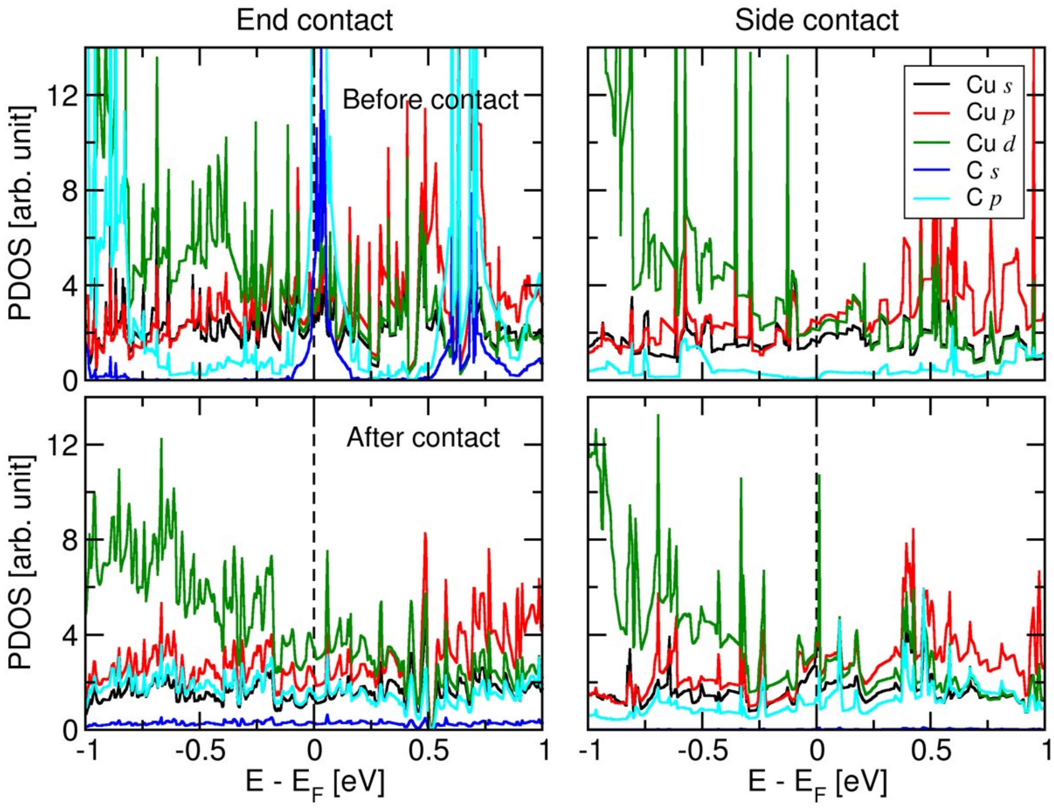

Figure S2. (Colour online) Partial density of states (PDOS) for copper (*s*, *p* and *d* orbitals) and carbon (*s* and *p* orbitals) at a Cu {100}/CNT (5,5) interface for both the end and side-contact configurations. The calculation performed using DFT/PBE calculations.

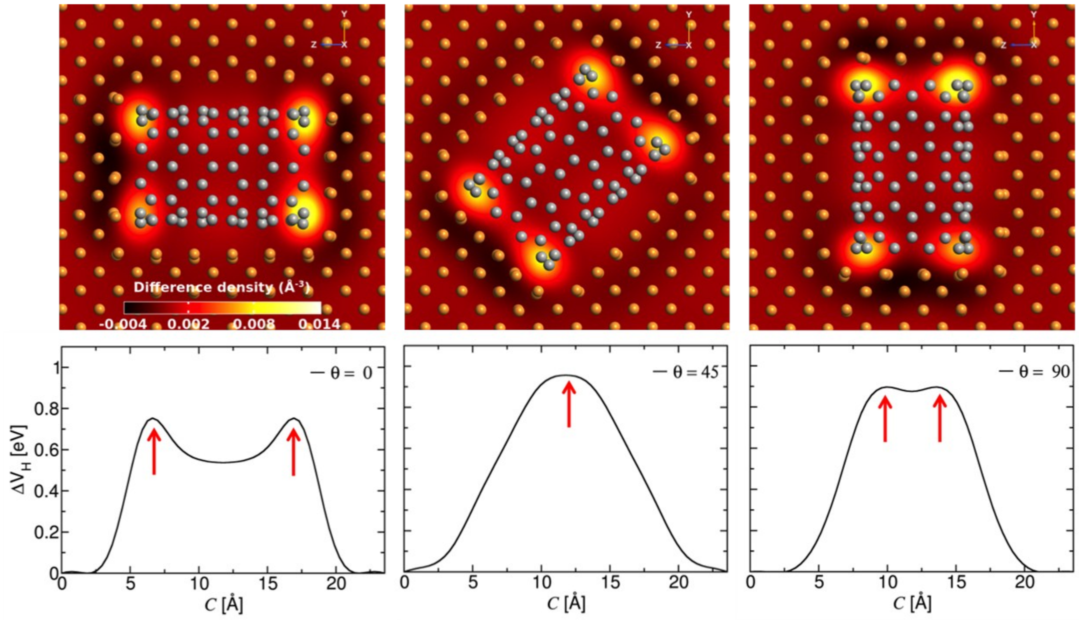

Figure S3. (Colour online) Electron difference density maps through cross-sections of the scattering region (upper) and the average electrostatic difference potential along the transport direction (lower) for the three (10,0) Cu-CNT composite orientations considered. The peak positions are indicated with red arrows to highlight the good correspondence between the upper and lower panel.

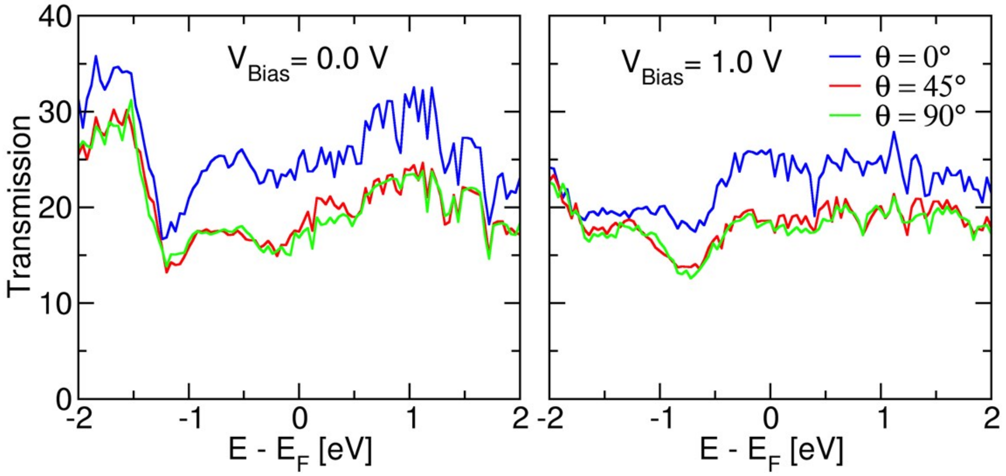

Figure S4. (Colour online) Transmission coefficients as a function of energy at V_{Bias} = 0.0 V and V_{Bias} = 1.0 V for (10,0) Cu-CNT composites with different values of θ. The Fermi level E_F is shifted to zero.

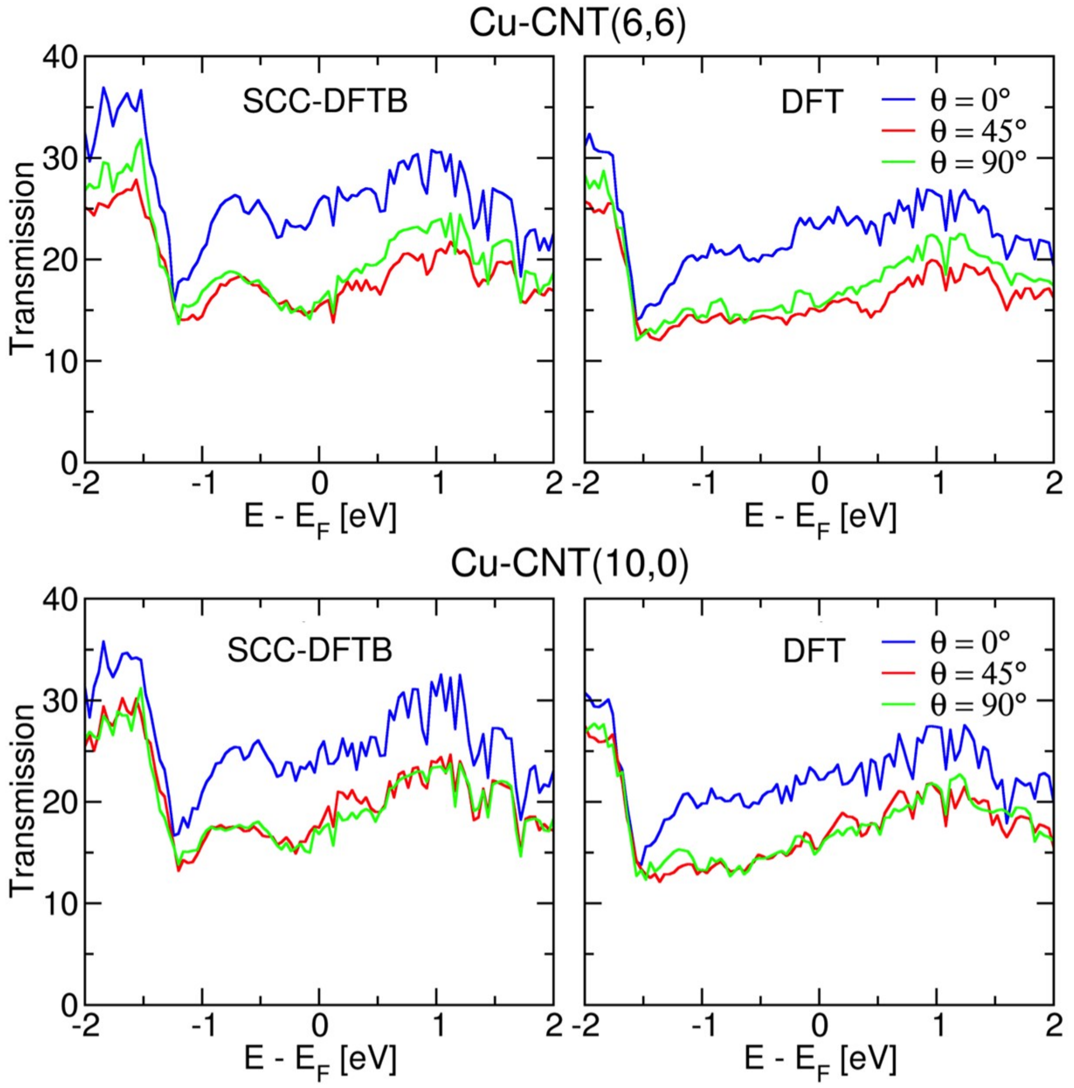

Figure S5. (Colour online) Transmission coefficients as a function of energy at V_{Bias} = 0.0 V obtained at SCC-DFTB and DFT level. The DFT results are obtained using a single-zeta plus polarization basis set and LDA functional. The Fermi level E_F is shifted to zero.

References:

(1) Perdew, J. P.; Burke, K.; Ernzerhof, M. Generalized gradient approximation made simple *Phys. Rev. Lett.* **1996**, *77* 3865–3868.

(2) Atomistix ToolKit 2014.0, QuantumWise A/S, www.quantumwise.com.

(3) Grimme, S. Semiempirical GGA-type density functional constructed with a long-range dispersion correction. *J. Comp. Chem.* **2006**, *27*, 1787-1799.

(4) Lin, Z.; Bristowe P.D. A first principles study of the properties of Al:ZnO and its adhesion to Ag in an optical coating. J. Appl. Phys. **2009**, *106* 013520.
